# Cardiorespiratory responses to 6-minute walk test in interstitial lung disease: not always a submaximal test

**DOI:** 10.1186/1471-2466-14-136

**Published:** 2014-08-11

**Authors:** Anne E Holland, Leona Dowman, Julio Fiore, Danny Brazzale, Catherine J Hill, Christine F McDonald

**Affiliations:** 1 Alfred Health, Melbourne, Australia; 2 La Trobe University Clinical School, Alfred Health, 99 Commercial Rd, Melbourne, VIC 3004, Australia; 3 Institute for Breathing and Sleep, Heidelberg, Australia; 4 Austin Health, Melbourne, Australia

**Keywords:** Exercise test, Pulmonary fibrosis, Lung diseases, Interstitial

## Abstract

**Background:**

The 6-minute walk test (6MWT) is used to measure exercise capacity and assess prognosis in interstitial lung disease (ILD). Although the 6MWT is usually considered to be a test of submaximal exercise capacity in ILD, the physiological load imposed by this test is not well described and 6MWT outcomes are poorly understood. This study aimed to compare cardiorespiratory responses to 6MWT and cardiopulmonary exercise test (CPET) in people with ILD.

**Methods:**

47 participants with ILD (27 idiopathic pulmonary fibrosis (IPF), mean age 71 (SD 12) years, diffusing capacity for carbon monoxide (TLCO) 49(15) %predicted) undertook CPET and 6MWT on the same day in random order. Oxygen uptake (VO_2_), ventilation (VE) and carbon dioxide production (VCO_2_) were assessed during each test using a portable metabolic cart.

**Results:**

The VO_2_peak during the 6MWT was lower than during CPET (1.17(0.27) vs 1.30(0.37) L.min^−1^, p = 0.001), representing an average of 94% (range 62-135%) of CPET VO_2_peak. Achieving a higher percentage of CPET VO_2_peak on 6MWT was associated with lower TLCO %predicted (r = −0.43, p = 0.003) and more desaturation during walking (r = −0.46, p = 0.01). The VEpeak and VCO_2_peak were significantly lower during 6MWT than CPET (p < 0.05). However, participants desaturated more during the 6MWT (86(6)% vs 89(4)%, p < 0.001). The degree of desaturation was not affected by the percent of peak VO_2_ achieved during the 6MWT. Responses were similar in the subgroup with IPF.

**Conclusions:**

On average, the 6MWT elicits a high but submaximal oxygen uptake in people with ILD. However the physiological load varies between individuals, with higher peak VO_2_ in those with more severe disease that may match or exceed that achieved on CPET. The 6MWT is not always a test of submaximal exercise capacity in people with ILD.

## Background

Exercise limitation is a cardinal feature of interstitial lung disease (ILD), resulting in reduced ability to undertake daily activities and poor quality of life [1]. Reduced peak oxygen uptake (VO_2_peak) [2-4] and exercise-induced hypoxemia [2,5] during the cardiopulmonary exercise test (CPET) are sensitive markers of mortality. Results from the CPET can also inform exercise prescription in people with ILD undergoing pulmonary rehabilitation. However, the CPET is not currently recommended as part of routine monitoring [6] and may not be available in all centres.

The six-minute walk test (6MWT) is a practical and inexpensive test of exercise tolerance that is commonly used to stage disease and evaluate treatment responses in people with ILD [2,7]. A reduced 6-minute walk distance (6MWD) is a predictor of mortality for people with idiopathic pulmonary fibrosis (IPF) in some [8,9] but not all [10] studies. Although the 6MWD has a significant relationship with other measures of outcome such as forced vital capacity (FVC) and diffusing capacity for carbon monoxide (TLCO) across a range of ILDs, these relationships are poor to modest in strength. [2,7,11,12] Some investigators have been reluctant to recommend the use of the 6MWT as it is unclear what it actually measures in people with ILD [13]. Whilst it is often considered a submaximal test, perhaps partially reflecting functional exercise tolerance [13], one previous study has suggested that the VO_2_peak achieved during a 6MWT may be equivalent to that during CPET in people with ILD [14], although this study may not have been sufficiently powered to detect differences between the tests.

It is possible that the 6MWT provides unique information that is not available from CPET. Oxyhemoglobin desaturation, a marked feature of the 6MWT in ILD, is a more consistent predictor of reduced survival than distance walked [2,15,16]. Data from people with other respiratory diseases have shown greater desaturation during walking compared to cycling [17], suggesting that the full extent of exercise-induced desaturation may only be visible during a walking test. However, an adequately powered comparison of the cardiorespiratory responses to CPET and 6MWT in ILD has not been undertaken.

The aims of this study were (1) to assess cardiorespiratory responses to the 6MWT in people with ILD; and (2) to compare cardiorespiratory responses with those obtained during CPET.

## Methods

Patients with documented ILD of any etiology were recruited from a tertiary hospital in Melbourne, Australia. Diagnosis was made according to established criteria [6,18]. Patients were eligible to participate if they were ambulant and able to ride a stationary cycle ergometer. Exclusion criteria were clinical instability, history of syncope on exertion and presence of comorbidities that precluded exercise testing (for example, orthopedic or neurological disease). Patients were also excluded if they had resting oxygen saturation (SpO_2_) <88% on room air, as supplemental oxygen was not used during the metabolic monitoring. Measurements of spirometry and TLCO were obtained to quantify disease severity. Right ventricular systolic pressure was estimated on transthoracic echocardiogram. The research protocol was approved by the hospital and university Human Research Ethics Committees and written informed consent was obtained from each participant prior to testing (H2008/03363, FHEC08-021).

### Study design and procedures

Participants performed the 6MWT and CPET in random order to offset the possible confounding effect of fatigue. All tests were performed in the morning and participants were permitted to take their regular medications prior to testing. Test order was concealed in a sealed opaque envelope until just prior to the first test. An interval of at least 60 minutes was provided between tests.

### Cardiorespiratory responses to exercise

Cardiorespiratory response to the tests was measured directly using a portable metabolic monitoring system (MetaMax 3B, Cortex, Germany). This system weighs approximately 570 grams and is designed to be worn on the chest with a harness. A turbine digital transducer measured inspired and expired airflow via a facemask, while an electrochemical cell oxygen analyzer and an infrared carbon dioxide analyzer simultaneously measured expired gases. Values of breath-by-breath oxygen uptake (VO_2_), carbon dioxide production (VCO_2_) and minute ventilation (VE) were averaged every 20 seconds. The system was calibrated before each test according to the manufacturer's specifications. Heart rate (HR) and rhythm were monitored through a 3-lead electrocardiogram (ECG). Data were recorded via telemetry, stored in an on-board memory and then downloaded to a computer for analysis.

Oxyhemoglobin saturation was monitored continuously during the tests using a portable pulse oximeter (Tuffsat, GE Healthcare, Finland). Baseline SpO_2_, nadir SpO_2_ (the lowest SpO_2_ during the test) and end-test SpO_2_ (the SpO_2_ at the point of test termination) were recorded. The modified Borg score (0–10 scale) was used to measure dyspnea and leg fatigue at baseline and at the end of each test.

### 6-minute walk test

The 6MWT was performed according to standardised criteria [19]. Participants were instructed to walk as far as possible along a 30-meter corridor for six minutes, with the aim of achieving their maximum possible walking distance in six minutes. Standardised instructions were provided and standardised encouragement was given each minute. Participants were permitted to stop and rest if required but were encouraged to continue walking as soon as they were able. To control for any learning effect, two tests were performed, separated by at least 30 minutes of rest. The best distance was recorded. The VO_2_peak during the 6MWT was defined as the highest of the 20-second averaged oxygen consumption measured between 2nd-6th minute.

### Cardiopulmonary exercise test

A symptom limited incremental protocol was performed on an electronically braked cycle ergometer (Corival V2, Lode BV, Netherlands). Participants were instructed to rest for two minutes and then cycle at between 50–60 rpm. Initial workload was 10 Watts (W). Thereafter, work rate was increased by 10 W increments each minute until the participant reached volitional or symptom limited exhaustion. This protocol was selected to ensure that the highest possible peak workload was achieved [20,21]. For the CPET, VO_2_peak was defined as the highest of the 20 second averaged oxygen consumption measured in the last minute of the test.

### Statistical analysis

We hypothesized that the VO_2_peak would be significantly higher during CPET. The sample size requirement for this study was calculated based on the results of our pilot data where VO_2_peak was 0.12 L.min^−1^ higher (SD of 0.29 L.min^−1^) than the 6MWT [22]. According to this calculation, a sample of 47 participants was necessary to provide a statistical power of 80% with a two-sided 0.05 significance level.

Statistical analysis was performed using SPSS software version 19.0 (Chicago, Illinois, USA). Metabolic response to the CPET and 6MWT was compared using paired t tests. One-way analysis of variance (repeated measures) was used to analyze the VO_2_ profile throughout the 6MWT. Relationships between important CPET outcomes (VO_2_peak and nadir SpO_2_) and 6MWT outcomes (6MWD, 6MWT VO_2_peak and 6MWT nadir SpO_2_) were examined using Pearson’s correlation coefficients (r) for normally distributed data. A pre-specified subgroup analysis was performed for participants with IPF. All data are expressed as mean (SD) unless otherwise stated. A p value of <0.05 was considered statistically significant.

## Results

Forty seven participants were included in the study. Twenty seven (57%) had IPF. Other diagnoses included: asbestosis (n = 8), connective tissue related ILD (n = 3), sarcoidosis (n = 4), non-specific interstitial pneumonia (n = 3) and hypersensivity pneumonitis (n = 2). The baseline characteristics of the participants are presented in Table 1. Participants with IPF had similar characteristics to those with other ILDs apart from a lower TLCO% predicted.

**Table 1:** Baseline demographic characteristics of participants

	**All**	**IPF**	**Non-IPF**
Sample, n	47	27	20
Age, years	71 (11)	74 (8)	67 (15)
Gender - Male	34 (77)	21 (78)	13 (65)
FVC, % predicted	74 (18)	73 (20)	75 (18)
TLCO, % predicted	49 (14)	44 (11)	55 (17)*
RVSP, mmHg	35 (15)	39 (18)	28 (5)
VO_2_peak, ml.kg^-1^.min^-1^	16 (4)	15 (4)	17 (4)
VO_2_peak, % predicted	68 (24)	64 (21)	74 (28)
6MWD, meters	429 (130)	414 (142)	448 (114)

Results are expressed as mean (SD) or n(%). ILD = interstitial lung disease, IPF = interstitial pulmonary fibrosis, FVC = forced vital capacity, TLCO = diffusing capacity for carbon monoxide, RVSP = right ventricular systolic pressure, VO_2_peak = peak oxygen uptake, 6MWD = 6-minute walk distance. *p<0.05 vs IPF subgroup.

**Figure 1:**
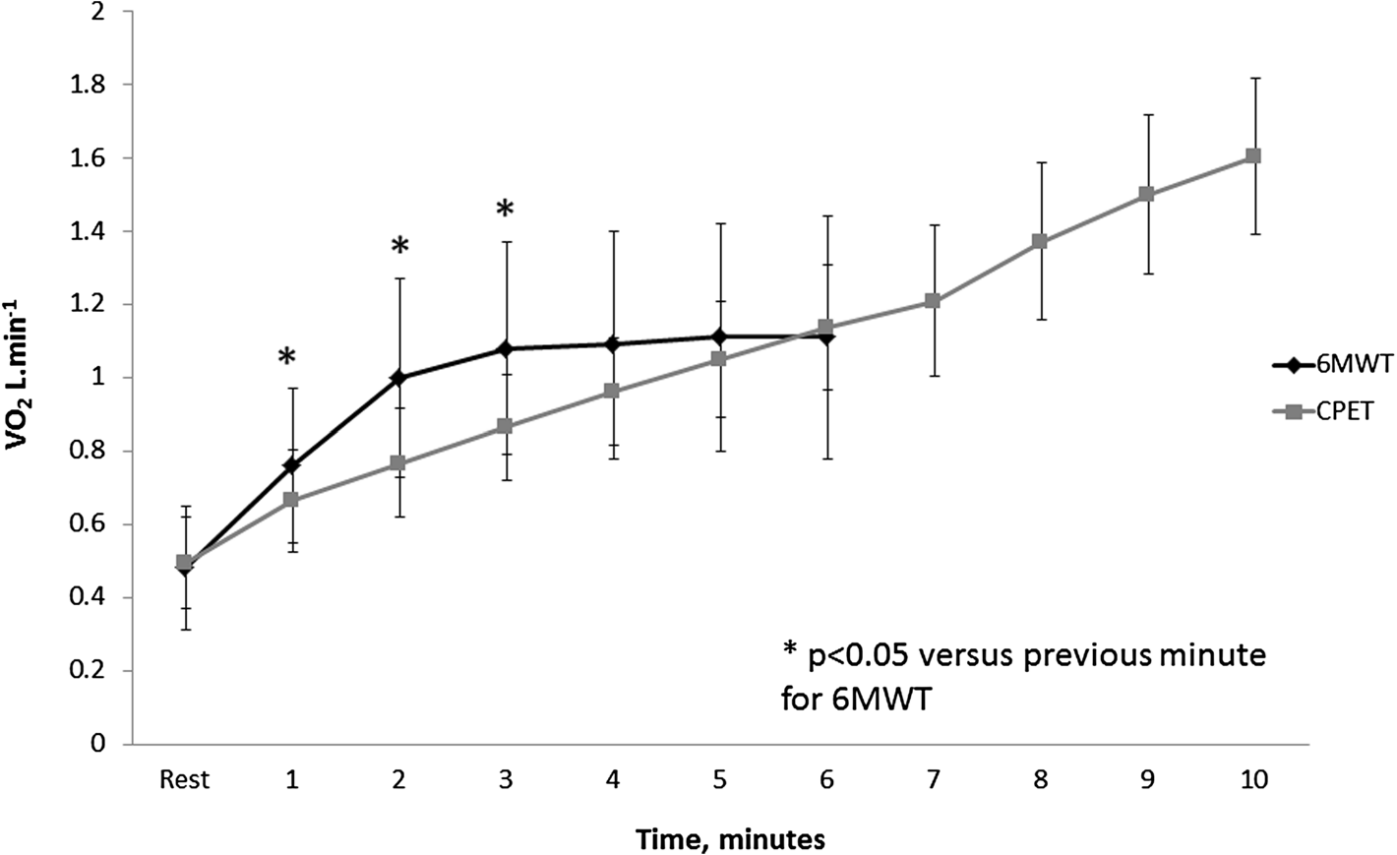
**Oxygen uptake (VO**_**2**_**) profile during the six-minute walk test.** Data are mean and standard deviation. * represents significant difference compared to previous minute on 6MWT.

Oxygen uptake increased progressively over the first three minutes of the 6MWT and achieved a plateau over the last three minutes (Figure 1). The same pattern of progression was observed in the subgroup of participants with IPF (0.77(0.23), 0.99(0.28) and 1.06(0.29) L.min^−1^ at 1, 2 and 3 minutes; p < 0.05 vs previous minute; 1.07(0.29), 1.08(0.29) and 1.1(0.29) L.min^−1^ at 4, 5 and 6 minutes, p > 0.05 versus previous minute).

The 6MWT VO_2_peak was significantly lower than that recorded during the CPET, averaging 94% of CPET VO_2_peak (p = 0.006 for comparison between exercise tests, Table 2). However there was wide variability between individuals, with 6MWT VO_2_peak ranging from 62% to 135% of VO_2_peak on CPET. Forty-five percent of individuals had a higher VO_2_peak during the 6MWT compared to the CPET. Eight participants stopped to rest during the 6MWT due to intolerable dyspnea, however the difference between tests persisted when these individuals were excluded from analysis (6MWT VO_2_peak 92% of CPET VO_2_peak, p < 0.001). The 6MWT was ceased due to dyspnea in 32 participants (68%) and due to leg fatigue in seven participants (15%), whilst the CPET was ceased due to dyspnea in 25 participants (53%) and due to leg fatigue in 11 participants (23%, p = 0.07 for comparison between tests).

**Table 2:** Comparison of cardiorespiratory responses to CPET and 6MWT

	**All (n =47)**			**IPF Subgroup (n = 27)**			**Non-IPF Subgroup (n = 20)**		
	**CPET**	**6MWT**	**p value**	**CPET**	**6MWT**	**p value**	**CPET**	**6MWT**	**p value**
VO_2_peak, L.min^−1^	1.25 (0.37)	1.16 (0.27)	0.006	1.19 (0.25)	1.15 (0.23)	0.20	1.46 (0.46)	1.22 (0.33)	0.002
VCO_2_peak, L.min^−1^	1.38 (0.43)	1.05 (0.30)	<0.001	1.36 (0.37)	1.05 (0.25)	<0.001	1.53 (0.52)	1.12 (0.34)	<0.001
VEpeak, L.min^−1^	55.5(16.6)	42.8 (14.2)	<0.001	56.8 (14.6)	47.2 (17.1)	<0.001	56.9 (20.6)	36.8 (12.1)	<0.001
HRpeak, bpm	127 (20.1)	118 (14.8)	0.001	126.1 (19.1)	118.3 (16.3)	0.009	128.6 (21.9)	118.5 (13.0)	0.03
NadirSpO_2_, %	89.3 (4.7)	86.5 (6.2)	<0.001	88.7 (5.4)	85.3 (6.2)	<0.001	90.2 (3.9)	88.1 (6.1)	0.07
Borg dyspnoea score	4.8 (1.6)	3.7 (1.3)	<0.001	5.1 (1.8)	4.0 (1.6)	0.006	4.6 (1.3)	3.5 (1.1)	<0.001
Borg fatigue score	4.1 (2.3)	3.0 (1.9)	0.001	3.9 (2.4)	2.5 (1.3)	0.004	4.4 (2.4)	3.7 (2.5)	0.13

Results are expressed as mean(SD). CPET = cardiopulmonary exercise test, 6MWT = six-minute walk test, VO_2_peak = peak oxygen uptake, VCO_2_peak = peak carbon dioxide production, VEpeak = peak minute ventilation, HRpeak = peak heart rate, NadirSpO_2_ = lowest oxyhaemoglobin saturation. p values are for comparison between tests.

**Figure 2:**
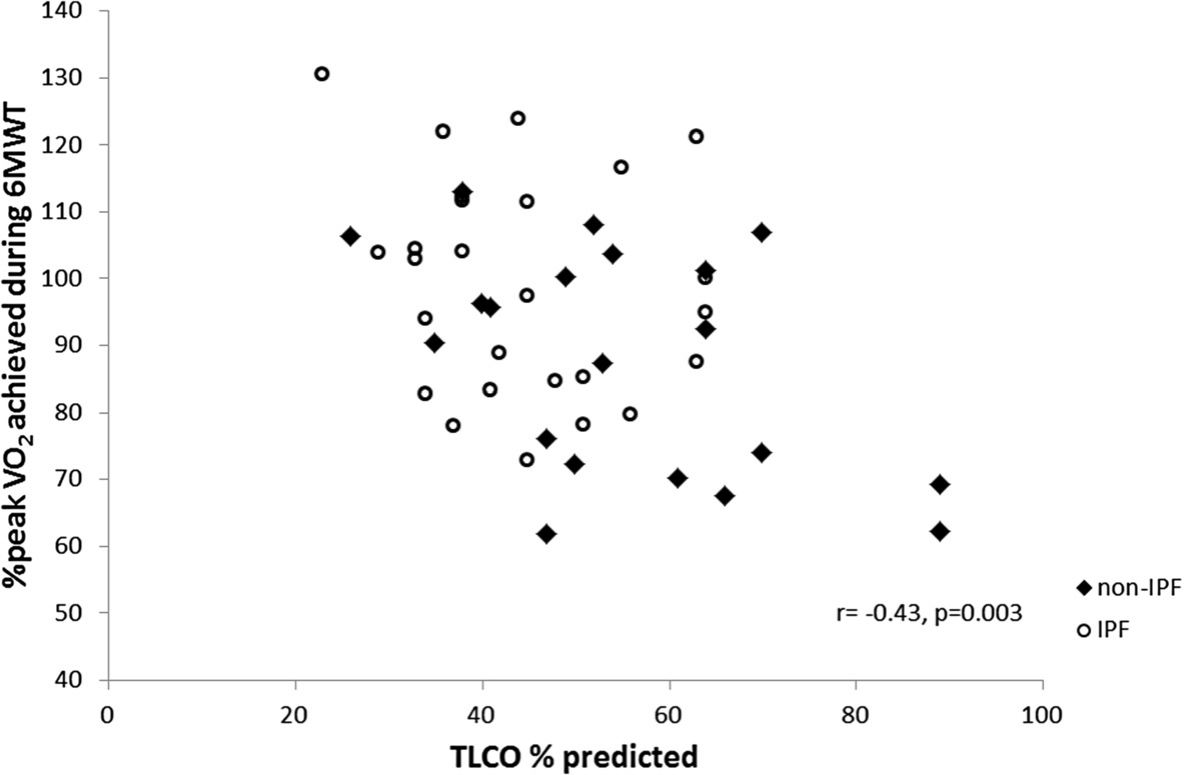
**Relationship between TLCO%predicted and % of peak VO**_**2 **_**achieved during 6MWT.**

**Figure 3:**
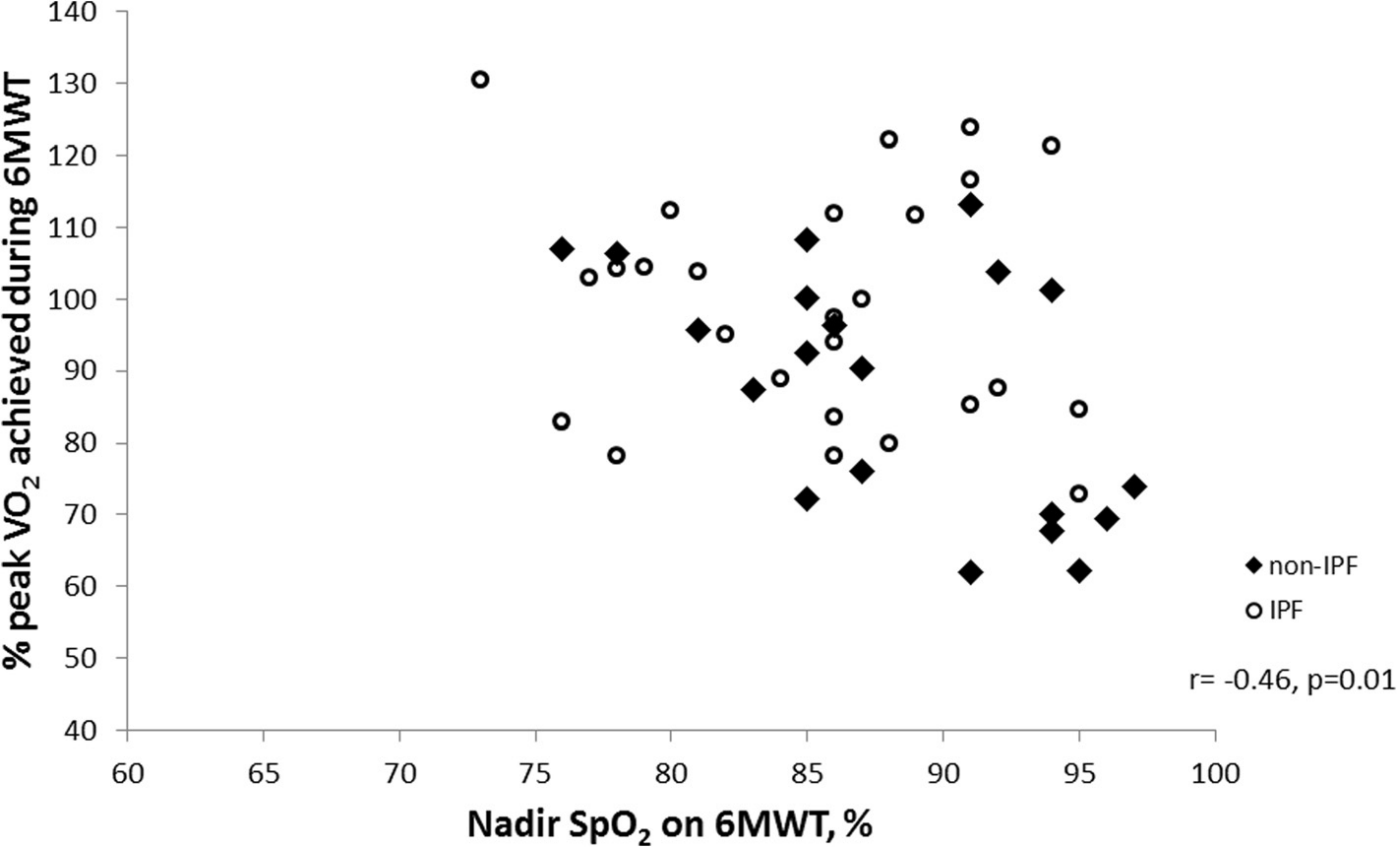
**Relationship between nadir SpO2 on 6MWT and % of peak VO**_**2 **_**achieved during 6MWT.**

The 6MWT VO_2_peak was a significantly higher percentage of CPET VO_2_peak in participants who had a lower TLCO%predicted (r = −0.43, p = 0.003, Figure 2) and greater desaturation during walking (r = −0.46, p = 0.01, Figure 3). There was no effect on the percentage of VO_2_peak achieved during 6MWT related to 6MWD, age, height, FVC, RVSP or percentage of peak HR achieved during walking. Other cardiorespiratory variables (VCO_2_peak, VEpeak and HRpeak) and scores of dyspnoea and fatigue were lower during the 6MWT in comparison to the CPET (p < 0.01). However, 37 participants (79%) desaturated more during the 6MWT than in the CPET (mean difference between tests 2.8%, p < 0.001). A similar pattern of differences in cardiorespiratory response was seen in the subgroup of participants with IPF, however the reduction in VO_2_peak on 6MWT was smaller and did not reach statistical significance (Table 2). Eighty-nine percent of participants with IPF desaturated more on the 6MWT than on the CPET (mean difference 3.4%, p < 0.001).

Longer 6MWDs were associated with a greater VO_2_peak during walking (r = 0.79, p < 0.001). Both the 6MWD and 6MWT VO_2_peak were strongly correlated with CPET VO_2_peak (r = 0.71 for both; p < 0.001) and with other CPET measures including peak work and VCO_2_peak (Table 3). However, the relationship of 6MWD and 6MWT VO_2_peak to other established measures of outcome and prognosis, such as respiratory function variables, was weak to moderate. In contrast, the nadir SpO_2_ on 6MWT had stronger relationships with prognostic variables such as FVC and TLCO%predicted and little relationship to other exercise responses including VO_2_peak. A similar pattern of results was observed in participants with IPF (Table 3).

**Table 3:** Relationship of 6-minute walk test outcomes to cardiopulmonary exercise test and demographics

	**All cohort (n =47)**			**IPF subgroup (n = 27)**			**Non-IPF subgroup (n = 20)**		
	**6MWD, meters**	**6MWT VO**_**2**_**peak, L.min**^**−1**^	**6MWT Nadir SpO**_**2**_	**6MWD, meters**	**6MWT VO**_**2**_**peak, L.min**^**−1**^	**6MWT Nadir SpO**_**2**_	**6MWD, meters**	**6MWT VO**_**2**_**peak, L.min**^**−1**^	**6MWT Nadir SpO**_**2**_
CPET VO_2_peak, L.min^−1^	0.71**	0.71**	0.28	0.74**	0.79**	0.24	0.55*	0.77**	0.35
CPET VCO_2_peak, L.min^−1^	0.77**	0.74**	0.19	0.77**	0.78**	0.14	0.69**	0.80**	0.35
CPET VEpeak, L.min^−1^	0.36*	0.21	−0.03	0.31	0.24	−0.37	0.46*	0.71**	0.29
CPET Peak Work, Watts	0.77**	0.38*	0.35*	0.86**	0.64**	0.15	0.73**	0.62**	0.48*
CPET NadirSpO_2_, %	0.21	0.13	0.76**	0.25	0.29	0.85**	0.05	0.03	0.61**
Age, years	−0.42**	−0.14	−0.23	−0.42*	−0.41*	0.04	−0.47*	−0.09	−0.35
Height, cm	0.09	−0.20	0.10	0.10	0.06	−0.15	0.15	0.51*	0.35
FVC%pred	0.22	−0.12	0.50**	0.13	0.04	0.49*	0.37	0.04	0.52*
TLCO%pred	0.34*	0.14	0.60**	0.28	0.35	0.64**	0.39	0.27	0.47*
RVSP, mmHg	−0.46*	−0.33	−0.48	−0.49	−0.43	−0.47	−0.16	0.37	0.73

Data are Pearson’s r. *p <0.05, **p < 0.01 for relationship between variables. CPET = cardiopulmonary exercise test, VO_2_peak = peak oxygen uptake, 6MWT = six-minute walk test, 6MWD = six-minute walk distance, NadirSpO_2_ = lowest oxyhaemoglobin saturation, VO_2_ – oxygen uptake, VCO_2_ - carbon dioxide production, VE – ventilation, FVC – forced vital capacity, TLCO – diffusing capacity for carbon monoxide, RVSP – right ventricular systolic pressure on trans-thoracic echocardiogram.

## Discussion

Our direct comparison of the cardiorespiratory responses during the 6MWT and CPET in people with ILD provides novel physiological data to explain the different performance characteristics of these important tests. This study has shown that, on average, the 6MWT elicits a high but submaximal VO_2_ in people with ILD that reaches a plateau after the third minute of the test. This supports the conceptualization of the 6MWT as a submaximal, steady state exercise test. However there is variability in responses that is related to disease stage. The 6MWT imposes a greater cardiorespiratory load on those with greater disease severity, where the peak VO_2_ can equal or exceed the VO_2_peak seen during a CPET. Oxyhaemoglobin desaturation was significantly greater during the 6MWT than the CPET and this was unrelated to the physiological load imposed by the test. These findings may assist in explaining the superior performance of oxyhaemoglobin desaturation compared to 6MWD as prognostic indicator in people with ILD.

It has previously been suggested that the self-paced nature of the 6MWT may contribute to the reduced predictive value of the 6MWD [3]. Our results show pronounced differences between 6MWT and CPET in VCO_2_peak and VEpeak, indicating a much lower physiological load for the 6MWT. This is similar to previous findings in COPD [23-26] and may reflect a smaller exercising muscle mass and greater lactate production during the CPET. More importantly, our results indicate that the 6MWT protocol frequently allows people with mild disease to exercise at a much lower percentage of their CPET VO_2_peak than those with more advanced disease. Not surprisingly, we found that a lower VO_2_peak was associated with lower 6MWD. This variability in the load imposed by the 6MWT across the range of disease severity may contribute to the inconsistency of 6MWD as a marker of prognosis across study populations [8-10].

In contrast, the degree of desaturation during exercise was unaffected by the VO_2_ elicited during the test. Although there was desaturation during both exercise tests, it was significantly greater during walking than during cycling for the vast majority of participants (Table 2). This is consistent with previous findings in COPD [17], which showed increased alveolar ventilation during cycling compared to treadmill walking, with a concomitant increase in partial pressure of oxygen in arterial blood (PaO_2_) which minimized the magnitude of oxyhaemoglobin desaturation during cycling. The increase in PaO_2_ during cycling compared to walking occurred prior to the anabolic threshold, which suggests that both neurogenic feedback from exercising muscles andearlier onset of anaerobic metabolism were contributors to better maintained oxyhaemoglobin saturation [17]. Whilst previous studies have found a consistent relationship between desaturation on 6MWT and poor prognosis, [2,15,16] the relationship between desaturation on CPET and prognosis is less consistent [2,3,27]. This may be because the lesser degree of desaturation on the CPET was not sufficient to discriminate those at risk of a poor outcome. Our data suggest that a greater degree of desaturation occurs during a 6MWT regardless of the physiological load imposed by the test, and this may be the most robust outcome of the 6MWT.

To date this is the largest study to compare the physiological responses to the CPET and 6MWT in individuals with any form of chronic lung disease. This may explain differences in our findings compared to others. One previous study included 13 individuals with ILD (12 IPF) and did not find a difference in VO_2_peak on 6MWT compared to CPET [14]. However, the small numbers of participants may have reduced the ability to detect small differences and did not allow the authors to assess effects attributable to disease severity. Their study also excluded people with pulmonary hypertension and thus the findings cannot be generalized to patients with more severe disease, where pulmonary hypertension is relatively common [28]. Studies which have examined the physiological load associated with the 6MWT in COPD have also found no difference in VO_2_peak between 6MWT and CPET [23-26]. This may represent a real difference in response across disease groups, or may reflect the smaller subject numbers in those studies (n = 12 to 26). It should be noted that we did not find a difference in mean VO_2_peak between tests when the subgroup of participants with IPF was examined separately (n = 27), however this study was not powered to assess differences in this subgroup and results should be interpreted with caution.

Other limitations to this study include conducting the exercise tests on the same day, such that fatigue may have impacted on results; however the order of testing was randomized to account for any potential order effects. Our exclusion criteria did not allow participation by patients with resting SpO_2_ of less than 88%, as supplemental oxygen was not applied during metabolic monitoring; our results therefore may not apply to patients with ILD and severe resting hypoxemia. Finally, a number of participants (n = 8) stopped during the 6MWT due to intolerable dyspnea, which occurs commonly in this group. This may have affected the physiological data collected and a steady state may not have been achieved. However, the difference between tests persists when these individuals are excluded from analysis.

## Conclusions

This study shows that the 6MWT elicits high but submaximal cardiorespiratory responses in people with ILD. There is wide variability across the range of disease severity, such that the 6MWT may elicit a higher VO_2_peak for those with more severe disease. Exertional desaturation is greater on 6MWT than CPET and this is unaffected by the physiological load. These data suggest that desaturation during a 6MWT may provide unique and consistent information across the range of disease severity in ILD.

## Abbreviations

6MWD: 6-minute walk distance; 6MWT: 6-minute walk test; CPET: Cardiopulmonary exercise test; FVC: Forced vital capacity; ILD: Interstitial lung disease; IPF: Idiopathic pulmonary fibrosis; RVSP: Right ventricular systolic pressure; SD: Standard deviation; TLCO: Diffusing capacity for carbon monoxide; VO2: Oxygen uptake; VE: Minute ventilation; VCO2: Carbon dioxide production.

## Competing interests

None of the authors have any conflict of interest to declare in relation to this manuscript.

## Authors’ contributions

AH conceived and designed the study, analysed and interpreted the data, drafted the manuscript and provided final approval of the manuscript. She had full access to the study data and is the guarantor of this manuscript. LD provided substantial contribution to acquisition of data, revised the manuscript critically for important intellectual content and provided final approval of the manuscript. JFJr analysed and interpreted the data, drafted the manuscript, revised the manuscript critically for important intellectual content and provided final approval of the manuscript. DB provided substantial contribution to acquisition of data, revised the manuscript critically for important intellectual content and provided final approval of the manuscript. CH contributed to study design, revised the manuscript critically for important intellectual content and provided final approval of the manuscript. CM contributed to study design, revised the manuscript critically for important intellectual content and provided final approval of the manuscript.

## Pre-publication history

The pre-publication history for this paper can be accessed here:

http://www.biomedcentral.com/1471-2466/14/136/prepub
